# Inclusion of endophenotypes in a standard GWAS facilitate a detailed mechanistic understanding of genetic elements that control blood lipid levels

**DOI:** 10.1038/s41598-020-75612-6

**Published:** 2020-10-28

**Authors:** Qianqian Zhang, Zexi Cai, Marie Lhomme, Goutam Sahana, Philippe Lesnik, Maryse Guerin, Merete Fredholm, Peter Karlskov-Mortensen

**Affiliations:** 1 Bioinformatics Research Centre (BiRC), Aarhus University, C.F.Møllers Allé 8, 8000 Aarhus C, Denmark; 2 Center for Quantitativ Genetics and Genomics, Aarhus University, Blichers Allé 20, 8830 Tjele, Danmark; 3 ICANalytics, Institute of Cardiometabolism and Nutrition (ICAN), 47-83 boulevard de l’hôpital, 75013 Paris, France; 4 Unité de Recherche sur les maladies cardiovasculaires, le métabolisme et la nutrition, INSERM UMR_S 1166, ICAN Institute of Cardiometabolism & Nutrition, Faculté de Médecine Sorbonne Université, Sorbonne Université, 4ème étage, Bureau 421,91, boulevard de l’Hôpital, 75634 Paris Cedex 13, France; 5 Animal Genetics, Bioinformatics and Breeding, Department of Veterinary and Animal Sciences, University of Copenhagen, Gronnegaardsvej 3, 1870 Frederikgsberg C, Denmark

**Keywords:** Genetics, Genetic association study

## Abstract

Dyslipidemia is the primary cause of cardiovascular disease, which is a serious human health problem in large parts of the world. Therefore, it is important to understand the genetic and molecular mechanisms that regulate blood levels of cholesterol and other lipids. Discovery of genetic elements in the regulatory machinery is often based on genome wide associations studies (GWAS) focused on end-point phenotypes such as total cholesterol level or a disease diagnosis. In the present study, we add endophenotypes, such as serum levels of intermediate metabolites in the cholesterol synthesis pathways, to a GWAS analysis and use the pig as an animal model. We do this to increase statistical power and to facilitate biological interpretation of results. Although the study population was limited to ~ 300 individuals, we identify two genome-wide significant associations and ten suggestive associations. Furthermore, we identify 28 tentative associations to loci previously associated with blood lipids or dyslipidemia associated diseases. The associations with endophenotypes may inspire future studies that can dissect the biological mechanisms underlying these previously identified associations and add a new level of understanding to previously identified associations.

## Introduction

Blood lipid levels are routinely measured to evaluate risk of cardiovascular disease (CVD), which is a devastating disease in humans. According to WHO, 17.5 million people died from CVD in 2012. This was more than twice the number of deaths due to cancers. Furthermore, six million people were under age 70 as CVD caused a premature end of life^1^. This places CVD as the leading cause of death and early demise in the world. The culprit of CVD is dyslipidemia, and this emphasizes why it is of crucial importance to understand the mechanisms that regulate serum levels of blood lipids.

Whole body cholesterol content and serum levels of cholesterol are regulated by a balance in uptake of cholesterol from food, excretion of cholesterol via the bile, de novo biosynthesis of cholesterol in the body as well as microbial metabolism and reduction of cholesterol to coprostanol^2,3^. Several genes and molecules that facilitate and regulate uptake, excretion and fluxes of cholesterol between compartments and over cell membranes have been described^4–8^. De novo biosynthesis of cholesterol can take place in any cell in the body, but the great majority of the molecule is synthesized in the liver in a tightly regulated process^9^. The homeostatic mechanisms are however only partly understood.

Two separate pathways for cholesterol biosynthesis have been described; the Bloch pathway (BPW)^10^ and the Kandutsch-Russel pathway (KRPW)^11^. The initial steps in cholesterol synthesis are shared by both pathways. HMG-CoA reductase (the target of current cholesterol-lowering statin therapy) is central in the early step of the cholesterol synthesis where mevalonate is formed from acetyl-CoA. Mevalonate is hereafter converted to squalene (Sq), which again is turned into lanosterol (Lan), which has the cyclic nature of cholesterol. Lan is the starting point for both the BPW and KRPW. In the BPW, Lan is converted to cholesterol via an intermediate called desmosterol (Des), whereas cholesterol is synthesized from Lan via an intermediate called lathosterol (Lat) in the KRPW.

A fundamental regulatory mechanism for de novo synthesis involves sensing of intracellular cholesterol levels by the SREBP proteins (sterol regulatory element binding protein 1 and 2). If cholesterol is present, SREBP binds to SCAP and INSIG-1. Else, INSIG-1 dissociates from the complex and allows cleavage of SREBP, which is then free to migrate to the nucleus where it acts as a transcription factor for a number of genes including the LDL-receptor gene (*LDLR*) and the HMG-CoA reductase gene (reviewed by REF^12^). Up-regulation of the LDL-receptor increases uptake of low-density lipoprotein cholesterol (LDL-C) from the blood and up-regulation of the HMG-CoA reductase gene increases de novo biosynthesis of cholesterol. The SREBP proteins regulate the initial steps of cholesterol synthesis. Regulatory mechanisms later in the cholesterol biosynthesis are not as well characterized. It has been observed in mice that relative use of the BPW or the KRPW is tissue specific, but the mechanisms, that regulate relative use of the two pathways, are unknown^13^.

The present genome-wide association study (GWAS) is designed to dig deeper into the uncharacterized regulatory mechanisms by the use of intermediate phenotypes—also known as endophenotypes.

Use of endophenotypes in genetic mapping have been advocated since the beginning of the century^14^. The rationale is that an intermediate phenotype is more proximal to the direct effect of a single gene. Hence, an endophenotype is expected to be genetically less complex than an end-point phenotype. This has implications both for the power of a GWAS study and for the interpretation of GWAS results into biological function. A GWAS detects the effect of a variation in the genome—most often a single nucleotide polymorphism (SNP). Associating a SNP with an appropriate endophenotype determines the direct effect of the given SNP, whereas associating the SNP with an end-point phenotype determines a derived effect of the SNP. The derived effect may be influenced by other genes and/or environmental factors and the resulting noise cause a reduction in statistical power. The biological complexity of an end-point phenotype furthermore hampers the biological interpretation of an identified association because the associated SNP, or genes near the genetic variant, may affect one of many phenotype-determining factors. For the endophenotype, on the other hand, the associated SNP, or genes nearby, may affect one of few endophenotype-determining factors.

There has been a general realization that endophenotypes are required in order to resolve genetic heterogeneity^15^ and to enable a functional characterization of newly discovered genetic variants^16^. Nevertheless, efforts to increase statistical power in GWAS has until now mainly been focused on increasing sample sizes^17^. This has also been the case for GWAS studies on blood lipid levels and CVD in humans. Here, sample sizes have increased to several hundred thousand individuals^18–20^. However, the measured phenotypes in these studies are still the crude end-point phenotypes, LDL-C, HDL-C, TG levels, or a CVD diagnosis.

In the present study, we investigate the power of including endophenotypes in the analyses instead of increasing sample size. Furthermore, we use the pig, which is an established model for the highly prevalent human diseases involving lipid metabolism (metabolic syndrome, diabetes, obesity, and cardiovascular diseases)^21–23^. The pig offers an attractive alternative model to rodents because of their anatomical and physiological similarities to humans and the availability of genomic, transcriptomic, metagenomic, proteomic, and metabolomic tools for analysis of this species^24^. Regarding blood lipids, pigs transport most of their cholesterol in LDL-C, as do humans, while rodents carry the majority of cholesterol in high-density lipoprotein cholesterol (HDL-C) rather than LDL-C, making them less than desirable as models^25^. Additionally, high-cholesterol diets can induce human-like changes in the plasma lipoprotein profile of pigs, with ~ 60% of plasma cholesterol distributed in LDL particles. Currently, there is no single golden standard animal model of atherosclerosis and CVD development, but the pig is probably the best way to recreate human plaque instability that can be linked to hypercholesterolemia, obesity, and diabetes, which contributes to accelerated atherosclerosis^26,27^.

The goal of the present study is, like in other GWAS studies, to identify loci with an ultimate effect on cholesterol levels, TG levels and CVD. However, unlike previous studies, we include levels of intermediate metabolites in the cholesterol synthesis pathways as endophenotypes in the analyses. This includes serum levels of Lan as well as the BPW and KRPW specific intermediate metabolites, Des and Lat. Furthermore, serum levels of phytosterols and microbiota-derived sterols are included in the analyses. These sterols are absorbed from the gut by the same mechanisms as cholesterol. Hence, serum levels of these lipids provide an estimate for the efficiency of cholesterol absorption from the gut^28–30^. Our goal is to dissect the cholesterol phenotypes and thereby identify loci controlling specific steps and pathways for biosynthesis, catabolism, and cholesterol transport mechanisms. Hereby we will add to a deeper understanding of biological and genetic mechanisms in cholesterol regulation.

## Results

The examined endophenotypes and end-point phenotypes are listed in Table 1 and results of the GWAS analysis are summarized in Table 2. We identified two genome-wide (*p* < 4.8E−08) and 10 suggestive associations (*p* < 4.8E−07). As a general overview, six loci were found to be associated with levels of different precursor molecules in the cholesterol synthesis pathway (Lan, Des, Lat, and Sum of intermediates (Sint)). One of these was genome-wide significant. Two suggestive associations were identified for end-point phenotypes LDL-C and total cholesterol (TC). Three associations (1 genome-wide significant) were identified for levels of phytosterols; campesterol (Cste), betasitosterol (Bsit), and sum of phytosterols (Sphy). One suggestive association were identified for levels of the microbiota-derived sterol, coprostanol (Csta).

**Table 1:** Phenotypes.

Phenotype	Abbreviation	n	Min	Max	Mean	SD
***Endophenotypes***						
*Intermediates in cholesterol biosynthesis*						
Squalene	Sq	245	0.70	17.30	4.96	3.69
Lanosterol	Lan	284	1.43	5.89	3.42	0.91
Lathosterol	Lat	299	13.06	96.99	49.85	17.15
Desmosterol	Des	300	22.33	123.63	68.35	20.06
Sum of intermediates	Sint	300	36.15	222.51	129.05	35.00
*Phytosterols*						
Betasitosterol	Bsit	297	45.36	470.17	186.60	96.30
Campesterol	Cste	288	106.68	862.77	392.42	158.37
Stigmasterol	Stig	249	0.16	2.56	1.14	0.48
Sum of phytosterols	Sphy	290	187.96	1253.79	577.60	232.74
*Microbiota-derived sterols*						
Coprostanol	Csta	298	1.95	31.12	13.66	6.35
Epicoprostanol	Esta	276	0.23	5.41	2.23	1.11
Sum of microbiota-derived sterols	Sste	303	2.17	35.01	15.84	7.28
***End-point phenotypes***						
LDL-Cholesterol	LDL-C	296	30.82	86.53	57.70	10.67
HDL-Cholesterol	HDL-C	317	13.00	57.00	36.19	7.92
Total Cholesterol	TC	297	67.78	137.84	100.88	13.88
Triglycerides	TG	295	42.57	200.12	119.68	31.02

All endophenotypes were measured in µg/dl. End-point phenotypes were measured in mg/dl.

SD = standard deviation of the mean.

**Table 2:** Genome-wide and suggestively significant GWAS results.

Pheno-type	Chr	Lead-SNP BP	MAF	*P*	B	SE	Genomic region	Candidate gene	Position of functional candidate gene	Functional link	Region in strong LD with lead-SNP, r^2^ > 0.8
LDL-C	2	82,111,387	0.10	4.60E−07	− 6.78	1.34	Intron, *FCHO2*	*FCHO2*	82,117,891-82,248,407	The *FCHO2* gene product is involved in clathrin-mediated endocytosis of LDL-C^31^	81,958,868-82,859,296
Csta	3	15,599,531	0.08	7.30E−07	− 4.24	0.86	Intron, *CALN1*	n/a		n/a	15,580,136-15,637,077
Des	6	157,486,305	0.19	1.06E−11	− 13.58	2.00	Intron, *DHCR24*	*DHCR24*	157,483,794-157,519,059	*DHCR24* encodes the enzyme that converts desmosterol to cholesterol^32,33^	157,481,935-157,486,331
Sint	7	57,828,885	0.22	3.62E−07	18.54	3.64	Intron, *LINGO1*	n/a		n/a	57,707,845-60,000,893
Bsit	13	53,636,117	0.39	2.57E−07	− 41.50	8.05	Intergenic	n/a		n/a	53,062,778-54,254,135
Sphy	13	75,586,650	0.32	1.30E−08	− 106.55	18.74	Intergenic	n/a		n/a	75,586,650-75,723,225
Cste	13	79,228,431	0.47	2.58E−07	− 65.28	12.67	Intergenic	*FAIM* *FOXL2*	79,398,049-79,411,772 79,708,693-79,709,825	*FAIM* deficiency enhances SREBP signaling and promotes lipogenesis in liver^34^. *FOXL2* represses expression of Star, which controls cholesterol transport in mitochondria^35,36^	79,109,893-79,247,374
Sint	13	88,500,932	0.53	2.37E−07	15.30	2.96	Intergenic	*CP*	89,398,185-89,463,223	*CP* encodes ceruloplasmin. Ceruloplasmin concentrations are correlated with serum triglyceride and cholesterol levels^37^	88,087,429-89,821,031
Lat	13	88,852,202	0.50	3.38E−07	8.02	1.57	Intergenic	*CP*	89,398,185-89,463,223	Do	88,084,836-90,249,783
Des	13	89,367,223	0.48	4.67E−07	8.48	1.68	Intron, *HPS3*	*CP*	89,398,185-89,463,223	Do	88,084,836-91,010,648
TC	13	89,563,053	0.47	2.84E−07	4.65	0.91	Intergenic	*CP*	89,398,185-89,463,223	Do	88,084,836-89,993,057
Lan	15	127,265,666	0.20	8.43E−08	0.57	0.11	Intron, *NYAP2*	n/a	127,032,688-127,295,095	n/a	127,263,233-127,267,733

Summary data of lead-SNPs surpassing the genome-wide (*p* < 4.8E−08) or suggestive (*p* < 4.8E−07) significance level. Results are listed in chromosomal and positional order. Chromosome number (Chr) and base-pair (BP) position refer to pig genome assembly Sscrofa 11.1. MAF = minor allele frequency. *P* = *p* value. B = regression coefficient. SE = standard error for B. r^2^ = The squared correlation coefficient between pairs of SNPs as a measure for linkage disequilibrium (LD). Phenotype abbreviations: Serum levels of the following lipids, Lanosterol (Lan), Lathosterol (Lat), Desmosterol (Des), Sum of intermediates in the cholesterol synthesis pathway (Sint) i.e. Lan + Lat + Des, Betasitosterol (Bsit), Campesterol (Cste), Stigmasterol (Stig) Sum of phytosterols (Sphy) i.e. Bsit + Cste + Stig, Coprostanol (Csta), low-density lipoprotein cholesterol (LDL-C), total cholesterol (TC).

Six of the genome-wide or suggestively associated SNPs were located in introns of protein-coding genes and six were within intergenic regions. Strong functional candidate genes were found for seven of the associated SNPs and one locus appeared to have a pleiotropic effect.

**Figure 1:**
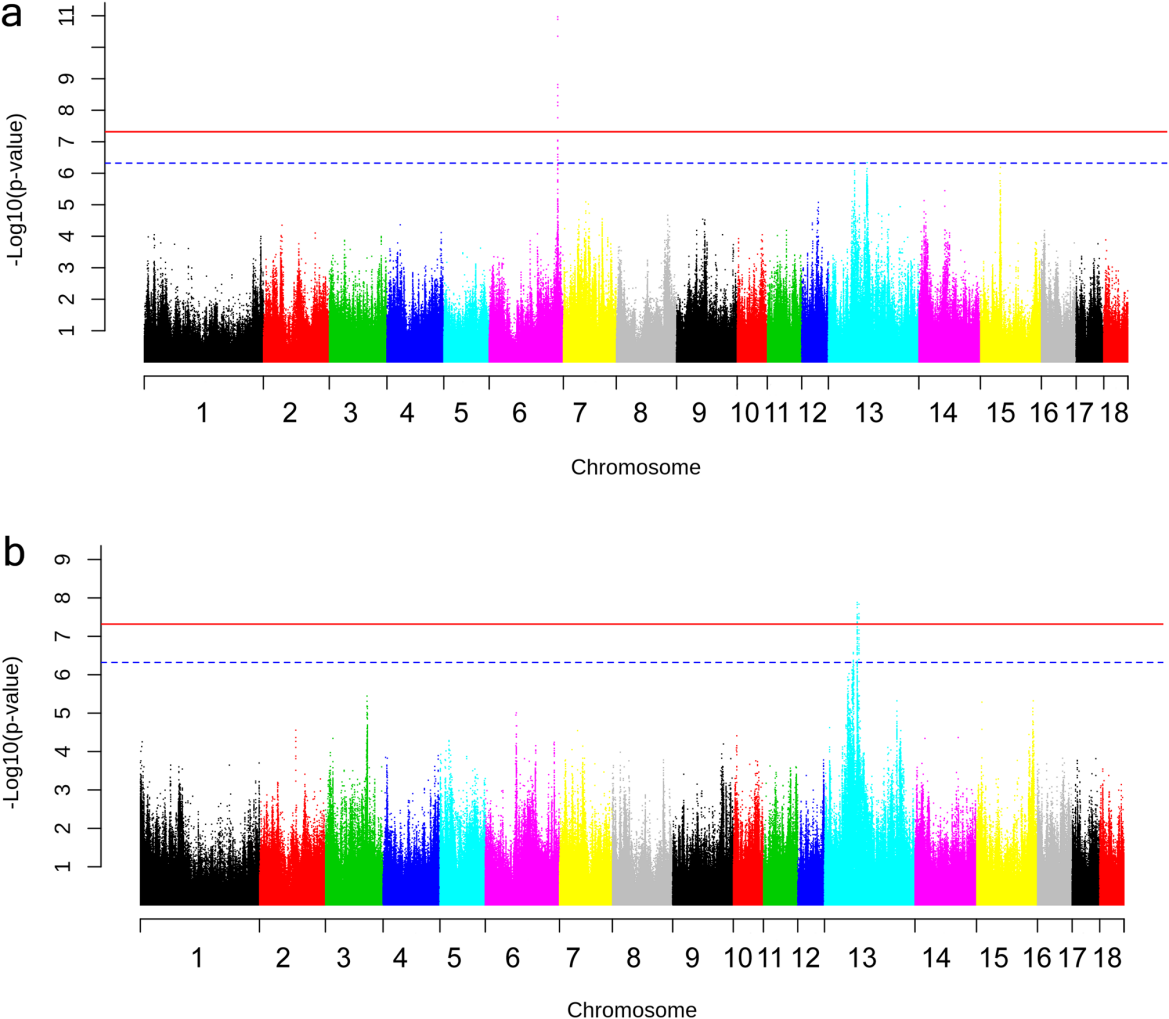
Manhattan plot illustrating results of the genome-wide association study for the phenotypes (**a**) Desmosterol (Des) and (**b**) Sum of phytosterols (Sphy). Chromosome numbers are indicated on the X-axis and − log(*p* values) on the Y-axis. Horizontal red line: Genome-wide significance threshold (− log(4.8E−08). Horizontal dashed blue line: Suggestive significance threshold (− log(4.8E−07).

**Figure 2:**
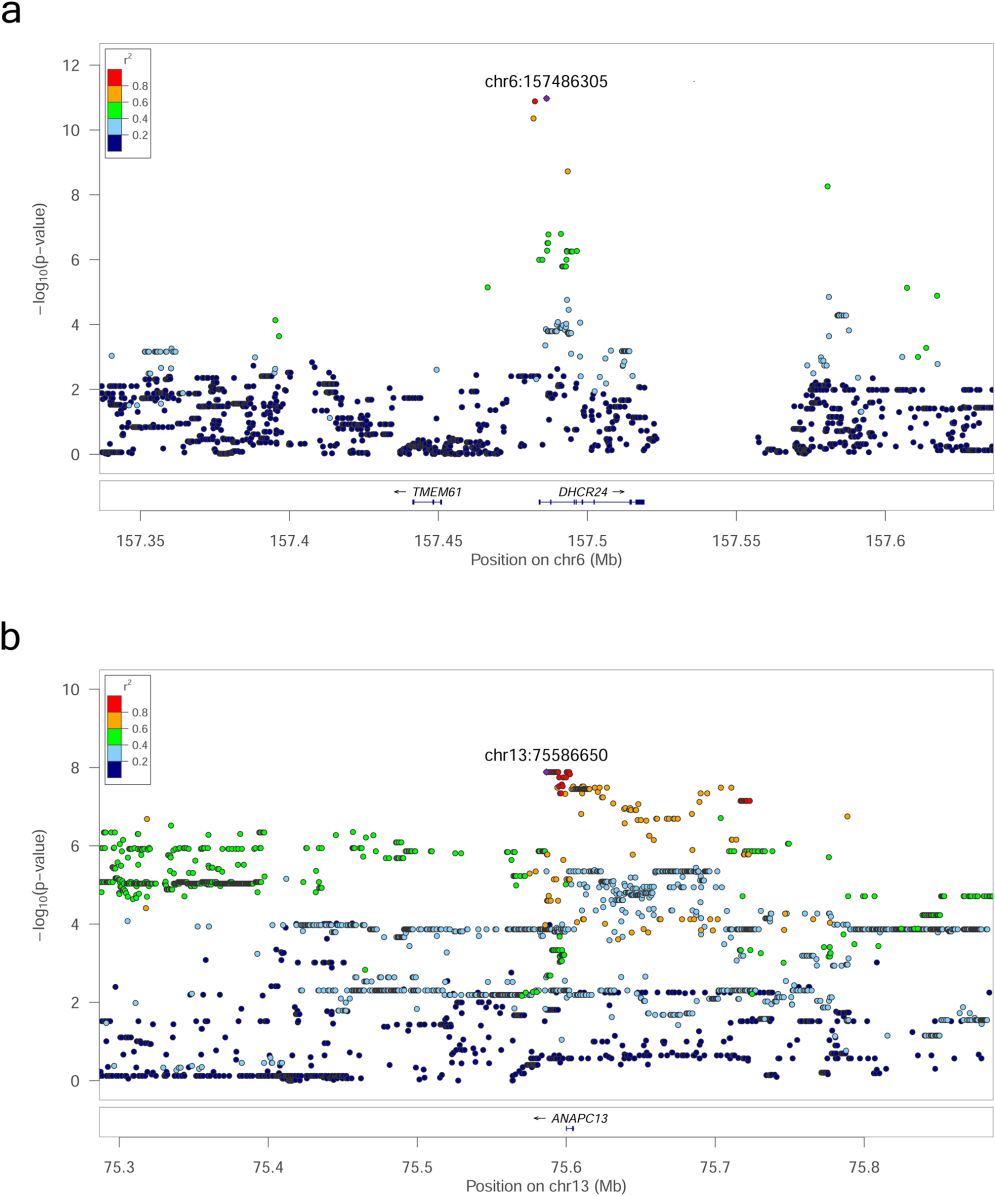
LocusZoom plots of regional genome-wide association results, linkage disequilibrium and gene annotation for the two genome-wide significant associations. (**a**) Cover the region on chromosome 6 associated with serum levels of desmosterol. (**b**) cover the region on chromosome 13 associated with sum of phytosterols in serum. Genome positions are indicated on the X-axes. Annotated genes in the regions are indicated above the X-axes; exons and introns are indicated by boxes and lines, respectively. − log(*p* values) are indicated on Y-axes. The squared correlation coefficients (r^2^) as a measure of linkage disequilibrium between SNPs are indicated by color. SNPs with r^2^ > 0.8 are considered in strong linkage disequilibrium.

The association between Des and a SNP on chromosome (Chr) 6, 157,486,305 bp was the most significant association identified (*p* = 1.06E−11) (Fig. 1a). The markers in strong linkage disequilibrium (LD) with this SNP position encompass a region of 4397 bp entailing exon 1 of *DHCR24* (Fig. 2a). This gene encodes the enzyme 24-dehydrocholesterol reductase that converts desmosterol to cholesterol and different mutations in this gene has been shown to cause desmosterolosis (OMIM:602398; Refs.^32,33^). Our finding shows that the limiting step for Des serum levels in the examined pigs are not synthesis of the molecule but rather conversion of the molecule to cholesterol by DHCR24.

The four phenotypes Lat, Des, Sint and TC were found to be suggestively associated with four different SNPs on Chr 13 in strong LD with each other. The Des-associated SNP was located in an intron of the *HPS3* gene but the remaining three SNPs were located in intergenic regions. All of them were located close to the *CP* gene, which encodes ceruloplasmin. Ceruloplasmin concentrations has previously been shown to be strongly correlated with serum triglyceride and cholesterol levels^37^. Furthermore, ceruloplasmin administration has been shown to produce a partial correction of dyslipidemia, manifested by normalization of levels of TG, TC, LDL-C and HDL-C^38^. A mechanistic explanation for this may be found in the ability of ceruloplasmin to oxidize LDL particles (reviewed by Ref.^39^). The oxidized particles are scavenged and degraded by macrophages (reviewed by Ref.^40^) and thereby ceruloplasmin has a LDL-C lowering effect.

LDL-C level was furthermore suggestively associated with a SNP on Chr 2 in an intron of *FCHO2*. The FCH and mu domain containing endocytic adaptor 2 encoded by *FCHO2* plays a direct role in clathrin-mediated endocytosis of LDL-C by organizing clathrin-coated structures for LDLR endocytosis^31^. Hence, this gene plays a role in clearance of LDL-C from the blood stream.

Sint and Lan were found to be suggestively associated with positions in introns of *LINGO1* on Chr 7 and *NYAP2* on Chr 15, respectively. To our knowledge, this is the first time these two genes has been linked to levels of cholesterol precursors in the blood, or blood lipid levels in general.

Three SNPs were associated with levels of different phytosterols. These results establish the first identified loci associated with serum levels of phytosterols. The three SNPs were located on Chr 13 but not close to each other. The Sphy associated SNP at 75.59 Mb on Chr 13 was genome-wide significant (*p* = 1.3E−08) (Fig. 1b) and the *ANAPC13* gene, encoding anaphase promoting complex subunit 13, was located within a region in strong LD with the lead-SNP (Fig. 2b). This gene has not previously been associated with serum levels of phytosterols or other lipids. A SNP suggestively associated with Cste was located close to the genes *FAIM* and *FOXL2*. The two genes are not obvious candidate genes for phytosterol levels but they are involved in lipid metabolism. FAIM deficiency enhances SREBP signaling and promotes lipogenesis in liver^34^. FOXL2 represses expression of Star, a protein that controls cholesterol transport from the outer to the inner mitochondrial membranes^35,36^.

The microbiota-derived sterol, Csta, was suggestively associated with a SNP on Chr 3. The SNP was located within an intron of the *CALN1* gene. To our knowledge, it is the first time this locus has been associated with lipid absorption or lipid metabolism and the result needs to be confirmed by further studies.

We furthermore identified 52 SNPs with a *p* -value between 1E−05 and 4.8E−07. Associations at this significance level will inevitably contain many false positive results. Table 3 lists 28 of the 52 SNPs. These SNPs are located in loci, which previously have been linked to blood lipid levels and/or dyslipidemia associated comorbidities. We classify these SNPs as tentative associations. Our results suggest that the previously identified effects may be mediated via specific molecular mechanisms identified by the tentative associations with specific endophenotypes in our study. For example, the *ARNT2* (Chr 7) and the *TSPYL5* (Chr 4) genes have previously been associated with blood lipid levels, regulation of lipid metabolism and fatty acid synthesis^41–43^. In our results, these two loci are tentatively associated with Lan. Hence, our results point more precisely towards a function of *ARNT2* and *TSPYL5* in the early steps of cholesterol synthesis where Lan is formed from Sq. Similarly, previous studies have linked *PID1* (Chr 15) to HDL-C level^44^ and *PEX2* (Chr 4) to levels of HDL-C and TC^45^, whereas the tentative associations in the present study point more precisely towards a function of these loci in the KRPW for cholesterol biosynthesis because the two loci are associated specifically with Lat levels. On the other hand, the associations between Des levels and the two genes *ARV1* (Chr 14) and *ACSL1* (Chr 15) identify functions of these loci in the BPW of cholesterol biosynthesis. These loci were previously associated with levels of LDL-C, free cholesterol in circulation and accumulations of cholesterol in the liver^46,47^, whereas our tentative results point more precisely towards an effect on BPW cholesterol synthesis. More examples like these are described in Table 3. Overall, the tentative results in the present study suggest new hypotheses about the fundamental factors and mechanisms causing previously identified end-point phenotype associations. The tentative results of the present study may therefore form a foundation for future studies to clarify these mechanisms.

**Table 3:** Tentative associations in regions previously associated with blood lipid levels or dyslipidemia associated comorbidities.

Pheno-type	Chr	Lead-SNP BP	MAF	*P*	B	SE	Genomic region	Candidate gene	Position of functional candidate gene	Functional link	Region in strong LD with lead-SNP, r^2^ > 0.8
Sste	1	5,288,352	0.30	7.64E−06	− 3.20	0.72	Intron, *PACRG*	*PACRG* *PRKN*	4,967,800-5,465,157 5,698,508-6,731,132	*PACRG* and *PRKN* are located head to head and are co-regulated (Entrez gene). *PRKN* encodes parkin, which is a lipid-responsive regulator of cellular fat uptake^48^ and has an effect on lipid absorption from the gut^49^	5,288,352-5,288,352
TC	1	26,458,317	0.12	2.56E−06	5.29	1.12	Intergenic	*TNFAIP3*	26,473,985-26,489,555	The product of *TNFAIP1* is a suppressor of the ASK1 activation that plays a key role in development of non-alcoholic steatohepatitis^50,51^	26,313,479-26,469,377
LDL-C	1	26,864,925	0.19	6.60E−06	4.76	1.06	Intergenic	*TNFAIP3*	26,473,985-26,489,555	Do	26,864,925-26,875,955
Bsit	1	97,876,982	0.01	8.22E−06	− 161.68	36.26	Intron, *ZBTB7C*	*ZBTB7C*	97,610,258-98,018,149	The product of *ZBTB7C* changes transcription factor binding dynamics of SREBP-1C in mice^52^	97,872,191-97,877,224
HDL-C	1	261,632,218	0.10	5.24E−06	− 4.37	0.96	Intergenic	*DAB2IP*	261,659,799-261,859,833	*DAB2IP* is associated with risk of coronary heart disease in humans^53^ that is correlated with HDL-C levels^54,55^	261,632,218-261,653,928
TC	2	80,066,762	0.07	2.60E−06	− 7.83	1.67	Intron, *COL23A1*	*COL23A1*	79,766,160-80,137,598	*COL23A1* encode a collagen variant found in lipid rafts in cell membranes^56^, which are involved in cholesterol transport^57^	80,066,184-80,075,271
Sint	4	6,495,094	0.08	2.29E−06	26.14	5.53	Intergenic	*MIR30D*	6,948,669-6,948,747	*MIR30D* is negatively correlated with HDL-C levels and positively correlated with LDL-C levels^58^	6,198,095-6,774,519
Lan	4	39,414,823	0.04	7.52E−06	0.85	0.19	Intergenic	*TSPYL5*	39,362,978-39,367,404	Down-regulation of *TSPYL5* occur concomitantly with a reduction in cellular cholesterol and fatty acid synthesis and a decrease in total cholesterol and free fatty acid levels^42,43^	39,414,823-39,414,823
Lat	4	58,499,547	0.05	5.38E−06	14.81	3.26	Intergenic	*PEX2*	59,252,313-59,270,099	*PEX2* controls levels of HDL-C and TC^45^	58,499,547-58,515,494
TG	4	108,751,861	0.03	1.42E−06	− 34.01	7.05	Upstream variant, *RAP1A*	*RAP1A*	108,673,364-108,694,720	*RAP1A* regulates hepatic and plasma PCSK9, plasma TC and LDL-C in mice^59^	108,751,861-108,751,861
TC	4	119,813,955	0.13	9.39E−06	5.05	1.14	Intergenic	*SNX7*	119,162,676-119,255,839	*SNX7* is associated with LDL-C levels^60^	119,765,369-119,828,033
Csta	5	90,450,047	0.14	5.80E−06	− 3.24	0.71	Intergenic	*EEA1*	90,131,742-90,259,208	*EEA1* encodes an early endosome antigen, which is a core component of endosome docking^61^ and may play a role in transport of lipids between membrane compartments^62^	90,344,197-90,566,508
Sste	5	90,450,047	0.14	9.69E−06	− 3.63	0.82	Intergenic	*EEA1*	90,131,742-90,259,208	Do	90,344,197-90,566,508
Sphy	6	71,581,677	0.10	9.99E−06	− 136.25	30.84	Upstream variant, *DISP3*	*DISP3* *UBIAD1* *ANGPTL7* *MTOR* *MFN2*	71,584,051-71,640,673 71,419,574-71,431,237 71,350,921-71,356,683 71,286,991-71,412,884 72,027,996-72,056,434	*DISP3* encodes a sterol-sensing-domain-containing protein^63^ *UBIAD1* may be involved in cholesterol metabolism (Entrez Gene) *ANGPTL7* plays a role in lipid trafficking and metabolism^64^ *MTOR* encodes a SREBP1 regulator^65^ *MFN2* plays a role in regulation of cholesterol synthesis^66^	71,549,824-71,591,026
TG	7	8,862,047	0.29	1.31E−06	11.88	2.46	Intergenic	*EDN1*	8,752,082-8,758,348	Endothelin-1 encoded by *EDN1* inhibits *IRS-1* expression and IRS-1 activity^67^. IRS-1 is a determinant for HDL-C and TG levels^68^	8,862,047-8,863,301
Lan	7	49,354,803	0.22	4.27E−06	− 0.41	0.09	Intron, *ARNT2*	*ARNT2*	49,259,648-49,450,466	*ARNT2* is associated with blood lipid levels^41^	49,347,252-49,354,803
Cste	7	53,150,849	0.14	6.45E−06	− 83.75	18.57	Intron, *CRTC3*	*CRTC3*		*CRTC3* is associated with total cholesterol plasma levels^69^	53,038,983-53,169,774
LDL-C	8	131,059,165	0.13	6.10E−06	5.88	1.30	Upstream variant, *PKD2*	*SPP1*	131,077,825-131,085,327	*SPP1* regulates CYP7A1, which converts cholesterol to hydroxyl-cholesterol in the first step of bile acid synthesis^70^	131,057,567-131,064,130
TG	9	121,527,397	0.14	8.42E−06	16.21	3.64	Intron, *TOR1AIP1*	*TOR1AIP1*	121,515,664-121,566,795	*TOR1AIP1* encodes Torsin 1A interacting protein 1, which regulates hepatic VLDL secretion^71^	121,527,397-121,527,397
Sint	12	38,331,125	0.19	3.98E−06	− 16.31	3.54	Intergenic	*LHX1*	38,461,948-38,563,314	*LHX1* is associated with circulating lipid levels^72^	38,331,125-38,331,452
Des	12	38,656,438	0.16	8.52E−06	− 10.69	2.40	Intron, *ACACA*	*ACACA*	38,581,541-38,875,025	*ACACA* is involved in fatty acid synthesis^73^	38,656,438-38,723,182
TG	12	40,823,266	0.16	5.54E−07	− 17.69	3.53	Intergenic	*CCL2*	40,740,308-40,799,969	The *CCL2* gene has been associated with TG level, atherosclerosis and myocardial infarction^74^ and knock-out of *CCL2* in mice results in lower levels of cholesterol and TG^75^	40,823,120-40,850,200
Sint	14	12,003,949	0.27	1.02E−06	− 18.11	3.71	Intergenic	*PBK*	11,543,665-11,561,923	*PBK* interacts with cholesterol and modulate cell-signaling^76^	12,001,579-12,021,393
Des	14	59,484,064	0.09	3.56E−06	13.54	2.92	Intron, *TTC13*	*ARV1*	59,397,433-59,408,878	Decreased expression of *ARV1* cause hypercholesterolemia^47^	59,389,882-59,541,999
HDL-C	14	64,268,082	0.03	2.85E−06	7.58	1.62	Intergenic	*CDK1*	64,233,839-64,249,546	*CDK1* encodes a kinase, which stabilized SREBP1^77^	61,776,948-64,843,717
Des	15	45,876,346	0.04	6.44E−07	− 21.42	4.30	Intron, *ACSL1*	*ACSL1*	45,867,356-45,933,112	Hepatic *ACSL1* depletion causes a hypercholesterolemic phenotype in mice^46^	45,665,239-46,524,879
Bsit	15	93,370,432	0.02	3.23E−06	− 116.95	25.12	Intergenic	*COL3A1*	93,556,914-93,595,678	Expression of *COL3A1* is affected by plant sterols^78^	93,370,432-93,370,432
Lat	15	130,395,782	0.12	1.29E−06	9.42	1.95	Intergenic	*PID1*	130,079,133-130,332,658	*PID1 c*ontrols HDL-C level^44^	130,384,947-130,395,782

Tentatively significant results of the genome wide association analyses for loci, which has been associated with blood lipid levels and/or dyslipidemia associated comorbidities in previous studies and have a *p* value < 10E−5 in the present study. All associations are listed in chromosomal and positional order. Chromosome number (Chr) and base-pair (BP) position refer to pig genome assembly Sscrofa 11.1. MAF = minor allele frequency. *P* = *p* value. B = regression coefficient. SE = standard error for B. r^2^ = The squared correlation coefficient between pairs of SNPs as a measure for linkage disequilibrium (LD). Phenotype abbreviations: Serum levels of the following lipids, Lanosterol (Lan), Lathosterol (Lat), Desmosterol (Des), Sum of intermediates in the cholesterol synthesis pathway (Sint) i.e. Lan + Lat + Des, Betasitosterol (Bsit), Campesterol (Cste), Stigmasterol (Stig) Sum of phytosterols (Sphy) i.e. Bsit + Cste + Stig, Coprostanol (Csta), Epicoprostanol (Esta), Sum of microbiota-derived sterols (Sste) i.e. Csta + Esta, low-density lipoprotein cholesterol (LDL-C), high-density lipoprotein cholesterol (HDL-C), total cholesterol (TC), Triglycerides (TG). The tentative associations with endophenotypes in the present study point towards specific biological mechanisms, which may be the underlying causes for the observed associations with end-point phenotypes in previous studies.

Correlations between phenotypes are listen in Supplementary Table 1. In general, serum levels of intermediate molecules in the cholesterol synthesis pathway were correlated with each other, phytosterol levels were correlated with each other and levels of microbiota-derived sterols were correlated with each other. Despite the close functional relationship between endophenotypes and end-point phenotypes, correlations between these phenotypes were generally weak and non-significant. However, a moderate correlation was observed between Des and TC. Additionally, we observed a moderate correlation between Lan and the two phytosterols Bsit and Stig. LDL-C was more strongly correlated with TC than HDL-C.

## Discussion

Larger and larger cohorts has been the mantra for increasing power in GWAS for many years, and for a good reason of course. Results obtained repeatedly in large cohorts are more trustworthy than those, which are only supported by a few more or less coincidental observations in a small sample. However, in the incessant race for larger studies and big consortia, it seems the value of meticulous phenotyping has been comparatively neglected. The power of a study depends on the statistical significance criterion, the sample size and the magnitude of the effect that we want to detect. In a GWAS, the phenotypic effect of any nucleotide variant may be fixed but the accurate ascertainment of the phenotypic effect has consequences for the power of the study. At least two factors play a critical role for accurate measurement of the phenotypic effect. Firstly, any confounding environmental factor must be minimized. Secondly, the direct effect must be measured rather than a secondary or a derived effect, which may be genetically more complex and hence biased by other loci in the genome. In the present study, we minimized environmental effects by using the pig as an animal model and by raising the animals in a highly controlled environment including a uniform diet for all animals. Furthermore, in order to detect a more direct effect of genetic variants, we measured a number of endophenotypes, for example intermediate metabolites in the cholesterol synthesis pathway. On the other hand, opposing the trend towards larger studies, we reduced the number of animals in the study to a number, which must be considered an absolute minimum for a study of this kind.

Despite the very small sample size, our approach resulted in detection of two genome-wide significant associations with *p* -values of 1.06E−11 and 1.30E−08. While *p* -values like those are remarkable for a GWAS with this sample size, the many suggestive and tentative associations also show that the number of animals was too restrictive and statistical power suffered from that. Nevertheless, strong functional candidate genes were found for a large fraction of the suggestively and tentatively associated loci. Furthermore, many of those demonstrated how inclusion of endophenotypes could be used to dissect genetic and molecular mechanisms underlying more complex end-point phenotypes. Many of the loci associated with endophenotypes in the present study have previously been associated with the more complex end-point phenotypes HDL-C, LDL-C or a dyslipidemia-associated disease. In the previous studies, detection of the relatively weak effect of these loci on the end-point phenotypes was only possible because large cohorts were studied. The associations to end-point phenotypes were not confirmed for these loci in the present study, because it was underpowered to detect loci with weak effects on complex end-point phenotypes. Instead, by including endophenotypes we found these loci associated with some of the genetically less complex but biologically more fundamental mechanisms, which ultimately cause a change in levels of LDL-C, HDL-C or disease risk as documented by the previous large cohort studies. That is, we detected the same loci in a simpler setup and at a lower cost, and at the same time, we obtain a more detailed understanding of the biological mechanism by which the loci have an effect on the complex end-point phenotypes.

Inclusion of endophenotypes furthermore enabled identification of one pleiotropic locus on Chr 13 associated with Lat, Des, Sint, and TC. The pleiotropic effect indicates either a direct effect at several levels of cholesterol biosynthesis or an early and strong fundamental effect reflected in levels of intermediates in all later stages of cholesterol synthesis. Hence, due to inclusion of endophenotypes, this locus can be identified as a master regulator in cholesterol biosynthesis.

In addition to loci with effect on endophenotypes, the present study also identifies a number of loci with an effect on the end-point phenotypes, HDL-C, LDL-C, TC and TG. In several cases, these results point towards genes with a very central role in cholesterol synthesis or to loci with an effect on cholesterol clearance and/or excretion rather than synthesis. HDL-C level was tentatively associated with the *CDK1* gene, the product of which stabilizes members of the SREBP family^77^. As mentioned above, SREBPs play a key role in the biosynthesis of cholesterol^79^. LDL-C level was suggestively associated with a key mechanism for LDL-C clearance from the blood, namely LDLR endocytosis, via an association with the *FCHO2* gene. TC was tentatively associated with another transmembrane transport mechanism by its association with *COL23A1*. Full-length COL23A1 molecules are found in lipid rafts^56^, which are tightly packed microdomains of the cell membrane. Evidence suggests that these rafts are directly involved in reverse cholesterol transport^57^. Our results lend support to the cholesterol transporting capacity of COL23A1-containing lipid rafts and point to an important role in regulation of overall cholesterol levels. TG level was tentatively associated with *TOR1AIP1*, which plays an essential role in regulation of secretion of hepatic VLDL^71^, which is the principal carrier of TG in the blood. All these results are in agreement with the expectation that a study of this scale will only be able to detect the loci with strongest effect for the genetically more complex end-point phenotypes.

The serum levels of phytosterols and microbiota-derived sterols can be used as a proxy for the efficiency of sterol absorption from the gastrointestinal canal. Our results confirm previous observations of a role for the PACRG/PRKN locus in lipid absorption from the gut. Besides that, our results indicate that loci with a more general effect on lipid transport and biosynthesis also have a role to play in sterol absorption.

Overall, the present study corroborate many previous results from studies in human and mice and the great overlap between results affirm the quality of the pig as an excellent animal model for human blood lipid metabolism. Furthermore, we identify new loci associated with different blood lipid levels. These results must be further evaluated in future studies in humans and animal models. Most importantly, the study identifies suggestive and tentative associations between endophenotypes and genes, which previously have been associated with end-point phenotypes. These results suggest hypotheses about more fundamental molecular mechanisms underlying the previously identified associations with end-point phenotypes. We propose that these hypotheses should be evaluated in future studies in humans and in animal models. The study demonstrates how inclusion of endophenotypes has the power to detect biologically important loci even in a small-scale study. This is a cost-effective approach compared to larger GWAS with complex end-point phenotypes. At the same time, the results demonstrate how the inclusion of endophenotypes facilitates elucidation of specific details in biological mechanisms underlying variation in end-point phenotypes.

## Material and methods

### Animal material and sample collection

All animals used in the present study were a three-way cross between Duroc, Landrace and Yorkshire used in the Danish pig production system. The sows from crossings between Landrace and Yorkshire were inseminated with mixed semen from Duroc boars to produce the pigs used in the study. All parental animals were provided by Danbred (Herlev, Denmark). The pigs were produced in a production farm and raised under the conditions for production pigs in Denmark observing guidelines in the Danish “Animal Maintenance Act” (Act 432 dated 09/06/2004) and the “Order regarding animal experimentation” (BEK nr 12 af 07/01/2016) and approved by the Danish Veterinary and Food Administration. All pigs were ear-tagged with individual ID at weaning. Both female and male pigs were used in the study. All pigs were fed the same diet. None of the pigs was subjected to any treatment. At an age of approximately 6 month and a body weight of approximately 100 kg, animals were send to an approved commercial abattoir where they were slaughtered in the morning after overnight fasting. Blood and serum samples were collected immediately after exsanguination in BD K2E (EDTA) tubes and BD Vacutainer SST tubes, respectively, from Thermo Fisher Scientific.

### DNA isolation, genotyping and imputation to whole genome sequence variants

High quality DNA was isolated from EDTA stabilized blood using a classic salting out procedure^80^. SNP genotyping was performed by Edinburg Genomics, Ashworth Laboratories (Edinburgh) using the 700 K Affymetrix Axiom PigHD chip. To establish marker positions in the newest assembly of the pig genome (Sscrofa11.1^81^), sequences of the probes for Affymetrix Axiom PigHD SNPs were mapped to the new assembly using BWA^82^. Markers with a unique map position were retained for further analyses. Whole genome sequence (WGS) from a reference population of 217 animals from three pig breeds (89 Duroc, 61 Landrace and 67 Yorkshire) was used to impute genotypes at a WGS variant level based on HD chip genotypes. Non-autosomal markers and indels with a position coinciding with HD chip markers were removed. To phase the haplotype for the HD marker set and the WGS marker set, we used Eagle^83^ with default parameters. The WGS dataset comprised 26,581,741 bi-allelic markers on 18 autosomes. Finally, Minimac3^84^ was used to impute the HD marker set to WGS level. SNPs with a minor allele frequency below 2%, SNPs with large deviation from Hardy–Weinberg proportions (P < 1.0E − 4) and SNPs with imputation accuracy below 0.4, reported by Minimac3, were removed. After this, 14,763,710 markers were retained for association analyses.

### Phenotypes

Serum levels of all lipids and sterols listed in Table 1 were measured by Gas Chromatography-Mass Spectrometry (GC–MS) using a method adapted from Heuillet, et al.^85^ and Quehenberger, et al.^86^. Briefly, serum were supplemented with deuterium-labelled internal standards and esterified sterols were saponified with 500 µl 0.5 N KOH for 20 min at 60 °C. Sterols were extracted twice with 450 µl water and 900 µl hexane and derivatized with 60 µl BSTFA:TMCS (90:10), 1 h at 80 °C. Samples were dried and resuspended in 60 µl cyclohexane containing 1% BSTFA for GC/MS injection. Samples were analyzed using a Trace 1310-ISQ LT GC–MS instrument (Thermo Fisher Scientific). Sterols were injected at 250 °C in split mode and separated on a 30 m × 0.25 mm, 0.25 µm DB-5MS column (Agilent). Sterols were ionized using electronic impact (EI) and analyzed in SIM mode.

Outliers for each of the phenotypes were removed. A phenotypic value was considered an outlier if it was either below the first quantile − 1.5 IQR (interquartile range, which is the difference between third and first quantile), or above the third quantile + 1.5 IQR. The following linear mixed model was used to adjust the phenotypes for the fixed effects:

$${\varvec{y}}={\varvec{Z}}{\varvec{b}}+{\varvec{X}}{\varvec{g}}+{\varvec{e}}$$

where ***y*** is the vector of phenotypes; ***b*** is the vector of fixed effects (i.e. batch, pen, sex, group); ***g*** is the vector of random polygenic effect estimated using a genomic relationship matrix constructed using the markers; ***e*** is the random residual. It was assumed that ***g*** follows a normal distribution $N\left(0, G{\sigma }_{g}^{2}\right)$, in which ***G*** was the matrix of genomic relationship between individuals estimated using HD marker genotypes following VanRaden^87^, and ${\sigma }_{g}^{2}$ was the genetic variance. For random residuals, it was assumed that $e\sim N\left(0,I{\sigma }_{e}^{2}\right)$, where ${\sigma }_{e}^{2}$ was the residual variance and ***I*** was an identity matrix. The corrected phenotypes were calculated as estimated genetic values plus residuals $({y}_{c}=\widehat{g}+\widehat{e})$. These corrected phenotypes were used as the response variables in the association analysis.

Correlations between corrected phenotypes were calculated using the Hmisc R package (https://www.R-project.org). The False discovery rates (FDR) was calculated based on *p* -values using the *p*.adjust function of the R package.

### Genome wide association analysis

The method for identifying possibly closely linked QTL has been described previously^88^. We estimated the genomic relationship matrix (GRM) using GCTA^89^ with the 700 K HD marker set. We estimated the GRM for each chromosome by leaving this chromosome out. This GRM was used for the following GWAS analysis. SimpleM^90^ was used to estimate the number of independent tests and set a genome wide significance threshold *p*-value of 4.8e−08 (− log_10_P = 7.32). A threshold for suggestive significant was set to 10 times the genome wide significance level (4.8E−07). Furthermore, SNPs with − log_10_(*p *value) < 5 in regions, which previously had been associated with blood lipid levels or dyslipidemia associated comorbidities, are reported.

The GWAS was performed in several rounds. In a first round, single SNP GWAS analysis using GCTA^89^ was performed for each chromosome. Then all SNPs were ranked based on their − log_10_P value and the largest − log_10_P value within each chromosome was identified. If the − log_10_P value of a SNP exceeded five, and there were at least one SNP with − log_10_P > 4 within a 2 Mb region (1 Mb up and down stream of the lead SNP), this SNP was retained as lead-SNP. Then, for each lead-SNP we extracted the lead SNP’s genotype dosage, fitted it as a covariate, and scanned the whole chromosome again in a second round of the GWAS. If the result of second round detected another SNP that fulfilled lead-SNP criteria, and if this SNP had been a significant (− log_10_P > 5) non-lead SNP in the first round, then this SNP was added to the lead-SNP list. We then extracted the allele dosage of this SNP, fixed it as another covariate, and scanned the chromosome in a third GWAS round. This procedure was iterated until no additional SNP remained significant.

Genomic heritability (the proportion of phenotypic variance explained by all HD markers) was estimated for each phenotype using GCTA *--reml* function^89^ and a GRM with all HD markers on autosomes. LD was calculated between each lead-SNP and all other SNPs with − log_10_P > 5 on the same chromosome as the lead-SNP. Pairwise LD (r^2^) was calculated using Plink^91^. LocusZoom ^92^ was used to illustrate regional GWAS and LD results for genome-wide significant SNPs.

### Identification of candidate genes and comparative analysis with previous studies

For each identified association, a 1 Mb region of the porcine genome (Sscrofa11.1) centered on associated SNP were analyzed. Even though strong LD in many cases extended considerably less than 0.5 Mb on each side of the associated SNP, a 1 Mb region were analyzed for all associated positions. All protein coding as well as non-coding genes in the analyzed regions were catalogued. Previous knowledge about each gene was ascertained by searches in the PubMed database, the Entrez-Gene database and the database of www.genecards.org. Searches were performed with search terms combining each gene name with other relevant terms such as the specific endophenotype or end-point phenotype associated with the locus. For each gene, searches were also performed with a combination of gene name and the general search terms “sterols”, “cholesterol” and “blood lipids”.

## Supplementary information

Supplementary Table.
