# Supplementary material for: Inclusion of endophenotypes in a standard GWAS facilitate a detailed mechanistic understanding of genetic elements that control blood lipid levels
Source: Sci Rep. 2020 Oct 28;10:18434. doi: 10.1038/s41598-020-75612-6 (PMC7595098; doi:10.1038/s41598-020-75612-6)
Supplement: Supplementary file 1 — Supplementary Table. [file 41598_2020_75612_MOESM1_ESM.pdf]

Inclusion of endophenotypes in a standard GWAS facilitate a detailed mechanistic understanding of genetic elements that control blood lipid levels

Qianqian Zhang, Zexi Cai, Marie Lhomme, Goutam Sahana, Philippe Lesnik, Maryse Guerin, Merete Fredholm, Peter Karlskov-Mortensen

Supplementary Table 1: Phenotypic correlation between measured traits.

Correlation coefficients are presented in the upper right triangle and corresponding false discovery rates (FDR) are presented in the lower left triangle. Significant results are written in black. Insignificant results are written in grey. Phenotype abbreviations: Serum levels of the following lipids, Squalene (Sq), Lanosterol (Lan), Lathosterol (Lat), Desmosterol (Des), Sum of intermediates in the cholesterol synthesis pathway (Sint) i.e. Lan + Lat + Des, Betasitosterol (Bsit), Campesterol (Cste), Stigmasterol (Stig) Sum of phytosterols (Sphy) i.e. Bsit + Cste + Stig, Coprostanol (Csta), Epicoprostanol (Esta), Sum of microbiota-derived sterols (Sste) i.e. Csta + Esta low-density lipoprotein cholesterol (LDL-C), high-density lipoprotein cholesterol (HDL-C), total cholesterol (TC), Triglycerides (TG).

	Sq	Lan	Lat	Des	Sint	Bsit	Cste	Stig	Sphy	Csta	Esta	Ssta	LDL-C	HDL-C	TC
**Sq**		0	0.05	-0.05	0.14	-0.21	-0.15	-0.16	-0.2	-0.03	-0.04	-0.03	0.07	-0.05	0.04
**Lan**	1		0.46	0.39	0.51	0.35	0.1	0.32	0.23	0.13	0	0.12	0	0.06	0.13
**Lat**	1	2.15E-07		0.42	0.79	0.13	0.08	0.01	0.12	0.04	0.03	0.05	0.12	0.03	0.22
**Des**	1	7.24E-05	7.41E-06		0.85	0.13	0.17	0.22	0.18	-0.04	0	-0.04	0.18	0.25	0.37
**Sint**	1	3.69E-09	0	0		0.13	0.13	0.14	0.15	-0.01	0.01	0	0.18	0.17	0.35
**Bsit**	8.60E-01	1.04E-03	1	1	1		0.59	0.45	0.83	0.14	0.03	0.13	-0.03	0.02	0.03
**Cste**	1	1	1	1	1	1.00E-13		0.37	0.94	0.16	0.12	0.15	0.15	0.19	0.24
**Stig**	1	7.23E-03	1	5.31E-01	1	6.69E-07	2.55E-04		0.45	0.12	0.09	0.13	0.11	0.19	0.21
**Sphy**	1	4.50E-01	1	1	1	0	0	4.95E-07		0.17	0.09	0.16	0.09	0.15	0.18
**Csta**	1	1	1	1	1	1	1	1	1		0.58	0.99	-0.05	0.13	0.04
**Esta**	1	1	1	1	1	1	1	1	1	7.46E-13		0.67	-0.07	0.09	-0.02
**Ssta**	1	1	1	1	1	1	1	1	1	0	0		-0.05	0.13	0.04
**LDL-C**	1	1	1	1	1	1	1	1	1	1	1	1		0.12	0.8
**HDL-C**	1	1	1	1.78E-01	1	1	1	1	1	1	1	1	1		0.53
**TC**	1	1	6.26E-01	3.88E-04	8.05E-04	1	3.22E-01	1	1	1	1	1	0	3.13E-10	
